# Elucidating the Causal Relationships Between B Cells, Dendritic Cells, and Multiple Sclerosis Pathogenesis

**DOI:** 10.1002/brb3.71292

**Published:** 2026-02-28

**Authors:** Xuefei Wang, Yangwei Wang, Xiaojing Liu

**Affiliations:** ^1^ Department of Rheumatology and Immunology, Union Hospital, Tongji Medical College Huazhong University of Science and Technology Wuhan China; ^2^ Department of Thoracic Surgery, Union Hospital, Tongji Medical College Huazhong University of Science and Technology Wuhan China

**Keywords:** causal link, genome‐wide association study, human leukocyte antigen, immune cells, Mendelian randomization, multiple sclerosis

## Abstract

**Background:**

Multiple sclerosis (MS) is a chronic autoimmune disease that leads to significant disability, with its precise etiology still not fully understood. Recent studies indicate the crucial role of immune cells in MS pathogenesis. However, traditional research often focuses on major immune cell populations, potentially neglecting the roles of newly identified immune cell subsets with distinct receptors and functions. Moreover, conventional observational studies are prone to biases, hindering the establishment of definitive causal relationships. This study aims to investigate the causal relationships between 731 immune cell traits and MS susceptibility using Mendelian randomization (MR) analysis to provide more robust insights into the underlying mechanisms of MS.

**Methods:**

We performed MR analyses to assess associations between various immune cell traits and MS risk. Following this, we explored the molecular mechanisms of the significant associations, focusing particularly on B‐cell antigen presentation and the involvement of human leukocyte antigen (HLA) pathways.

**Results:**

Our analysis revealed specific causal links between B cells and dendritic cells with MS susceptibility. We identified 61 pleiotropic genes associated with MS. Notably, B‐cell antigen presentation and HLA‐related pathways play pivotal roles in MS pathogenesis. Additionally, alterations in immune cell populations post‐MS onset were observed, suggesting potential biomarkers for early diagnosis.

**Conclusion:**

This study offers a comprehensive examination of immune cell contributions to MS pathogenesis, identifying potential therapeutic targets and diagnostic markers. Given the side effects of anti‐B cell monoclonal antibodies in MS treatment, our findings propose avenues for more precise therapeutic strategies aimed at minimizing adverse effects.

## Graphical abstract

This study presents an integrative pipeline linking immune cell traits to multiple sclerosis (MS) susceptibility. Genome‐wide association and Mendelian randomization analyses revealed causal roles of immune cells in MS risk. Colocalization and pleiotropy mapping identified candidate genes, further refined through functional annotation, network analysis, and druggability assessment.

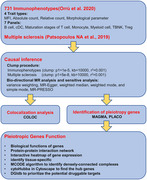

**Abbreviations**
APC: antigen‐presenting cell
BAFF‐R: B‐cell activating factor receptor
CSF: cerebrospinal fluid
EBV: Epstein–Barr virus
FDR: false discovery rate
GO: gene ontology
GWAS: genome‐wide association study
IMSGC: International Multiple Sclerosis Genetics Consortium
IVs: instrumental variables
IVW: inverse variance weighting
KEGG: Kyoto Encyclopedia of Genes and Genomes
LD: linkage disequilibrium
MAGMA: multi‐marker analysis of genomic annotation
MS: multiple sclerosis
MR: Mendelian randomization
OR: odds ratio
SNPs: single nucleotide polymorphisms

## 1 Introduction

Multiple sclerosis (MS) is an inflammatory demyelinating disease affecting the central nervous system, predominantly impacting young adults and leading to progressive neurological disability (Jakimovski et al. 2024). Severe physical torture and a high lifelong disability rate make it extremely urgent to explore the unknown etiology and find effective treatments for MS (Thompson et al. 2018).

Recent advances in MS research have highlighted the critical role of immune cells, including pro‐inflammatory T helper cells (Th1 and Th17) (Hu et al. 2017), CD8+ T cells (Clarkson et al. 2023), γδ T cells (McGinley et al. 2018), mast cells (Noto et al. 2021), and macrophages (Prinz et al. 2019). These findings underscore the heterogeneity of immune responses in MS. However, traditional tissue‐based studies offer static snapshots of established lesions, like inflammation and demyelination seen in postmortem tissue (Kuhlmann et al. 2017). The limited availability of early‐stage patient brain tissue and the narrow range of disease mechanisms covered by experimental models further restrict insights into disease onset (Vanderdonckt et al. 2022). Moreover, single‐cell transcriptomics offers valuable data on immune cell involvement but captures only time‐point observations and cannot establish causal relationships in disease progression (Kirschenbaum et al. 2024). Additionally, conventional observational studies are susceptible to biases, making it challenging to establish definitive causal relationships (Dahabreh and Bibbins‐Domingo 2024). As a result, it is necessary for a study to comprehensively consider multiple new populations of immune cell types and provide evidence regarding the causal association between immune cells and MS.

Mendelian randomization (MR) offers a robust approach to infer causality by utilizing genetic variants as instrumental variables (IVs), thereby mitigating confounding factors and reverse causation (Hemani, Zheng, et al. 2018; Davey Smith and Hemani 2014). Recent MR studies have explored associations between various diseases and MS, suggesting potential therapeutic targets (Patrick et al. 2023; Lin et al. 2023).

In this study, we adopt a comprehensive approach by analyzing 731 distinct immune cell traits to uncover potential links with MS. Leveraging genome‐wide association study (GWAS) data, we aim to identify specific immune cell abnormalities contributing to MS onset and elucidate the molecular mechanisms underlying these associations. Following this, we delve into the specific molecular pathways through which immune cells contribute to the development of MS. This detailed exploration not only enhances our understanding of the genetic basis of MS but also sheds light on the precise molecular events triggered by immune cell dysregulation.

## 2 Methods

### 2.1 Study Design

We evaluated the causal link between immune cell traits and MS using an MR methodology comprising two distinct sample groups. In MR, genetic variations serve as proxies for risk factors. Therefore, we carefully selected single nucleotide polymorphisms (SNPs) meeting three crucial assumptions, ensuring their validity as IVs. These genetic variants (1) are directly correlated with the exposure, (2) are not impacted by confounding factors, and (3) do not alter the result in ways other than through the exposure.

### 2.2 Source of GWAS Data for Immune Cells

The GWAS catalog's accession numbers, which range from GCST0001391 to GCST0002121, were used to retrieve the data (Orrù et al. 2020). The research investigated the impact of over 22 million genetic variations on 731 immune cell characteristics in a group of 3757 Sardinian people. This comprehensive analysis identified 459 independent and statistically significant association signals (*p* < 1.28 × 10^−11^) for 122 immune cell traits across 70 genomic loci, including 53 novel loci. These findings shed light on various molecular mechanisms governing cell regulation. Four Illumina arrays—OmniExpress, ImmunoChip, Cardio‐MetaboChip, and ExomeChip—were used for sample genotyping. Whole‐genome estimation was carried out regarding a panel of 3514 individuals from the Sardinian population, with adjustments made for gender, age, and age^2^ as covariates (Sidore et al. 2015).

### 2.3 Source of GWAS Data for MS

From the International Multiple Sclerosis Genetics Consortium (IMSGC), we obtained GWAS summary statistics for MS. This included information from 115,803 European–American individuals, including 47,429 cases and 68,374 controls (International Multiple Sclerosis Genetics Consortium 2019). Following rigorous quality control and imputation processes, a total of approximately 6.3 million genetic variants were subjected to analysis.

### 2.4 IV Selection

Each immune trait's significance threshold was set at 1 × 10^−5^, in line with recent research standards (International Multiple Sclerosis Genetics Consortium 2019; Ma et al. 2023). The significance threshold for the MS trait was established at 5 × 10^−8^. To begin, we employed European reference samples from the 1000 Genomes Project for aggregation processing. We applied stringent criteria, including a linkage disequilibrium (LD) threshold of *R^2^* < 0.001 and a window size of 10,000 kb. Subsequently, we retained only the SNP with the lowest *p*‐value (1000 Genomes Project Consortium 2015). We then calculated the *F*‐statistic for each IV and eliminated those with *F*‐values below 10, ensuring the use of robust IVs (Garfield et al. 2023). These carefully screened SNPs were employed in the subsequent stages of our analysis.

### 2.5 Colocalization Analysis

We employed the R‐package ‘COLOC’ for colocalization analysis (Giambartolomei et al. 2014) to determine whether there is a shared genetic causative SNP in a genomic region between immune cells and MS. For each immune trait, the most significant SNPs (lead SNPs) and their surrounding genomic regions were extracted based on GWAS summary statistics. Prior probabilities were set as *p*1 = 1 × 10^−4^, *p*2 = 1 × 10^−4^, and *p*12 = 1 × 10^−5^, following recommended practice for traits with polygenic architecture. This method is based on four hypotheses: H0 states that there is no discernible correlation between the two traits and SNP loci in the specific genomic region. H1 and H2 propose that SNP locations are associated with either Trait 1 or Trait 2 within the genomic area. H3 suggests that there is a substantial correlation between the trait and SNP loci in the specific genomic region, but the causative variation loci are different. Finally, H4 indicates a significant correlation between the trait and SNP loci in the specific genomic region, with the same causal variation locus for both traits.

### 2.6 Pleiotropy Analysis and Gene‐Based Association Analysis

To identify potential genes associated with both immune cells and MS, we employed multi‐marker analysis of genomic annotation (MAGMA) (de Leeuw et al. 2015). By leveraging data from the 1000 Genomes Project, specifically the European population data, MAGMA integrated LD between SNPs and identified multivariant effects, effectively establishing SNP‐to‐gene relationships. MAGMA provided *p* and *Z* values for each gene. A gene–phenotype association was considered significant if the false discovery rate (FDR) adjusted *p* value (*p*_FDR_) was less than 0.05.

To explore pleiotropy, we further applied the composite null hypothesis (PLACO) (Ray and Chatterjee 2020), which tests for the presence of a pleiotropic locus between the two traits, allowing for one locus to be linked to zero or one of the traits. PLACO utilized *Z*‐values to assess the correlation between the two phenotypes and a gene. This was based on four hypotheses: genes influencing the first characteristic but not the second; the second trait being correlated with genes, but not the first; the two disorders being unrelated to genes; and a gene being associated with both traits, indicating a pleiotropic connection (*p*_FDR_ < 0.05). Ultimately, a total of 61 genes were identified.

Notably, the two methods complement each other. MAGMA summarizes SNP associations at the gene level, which is well‐suited to detect cumulative polygenic effects but may miss signals driven by only a few variants. PLACO, in contrast, evaluates pleiotropy at the locus level and is more sensitive for identifying shared causal variants, although it may overlook broader polygenic contributions.

### 2.7 Enrichment Analysis and Tissue‐Specific Analysis

To conduct the Kyoto Encyclopedia of Genes and Genomes (KEGG) and gene ontology (GO) enrichment analysis on the 61 identified genes, we utilized the software program “clusterProfiler” (Wang et al. 2023). Additionally, we employed the “ggplot2” and “enrichplot” software tools for visualizing the enrichment results. Furthermore, we performed tissue‐specific analysis using the GENE2FUNC function of the FUMA web tools (Watanabe et al. 2017).

### 2.8 Protein–Protein Interaction (PPI) and Pathway Enrichment Analysis

We created a PPI network using 61 proteins and a STRING database (https://string‐db.org/) (confidence score cutoff = 0.4). Using the default settings, the MCODE cluster tool in Cytoscape (Shannon et al. 2003) was used to find the primary clusters among 61 protein interaction networks. Hub genes were identified by cytoHubba analysis in Cytoscape. The results were displayed as a simplified network with labels that corresponded to previously discovered functional enrichment. Using the Drug Gene Interaction Database (DGIdb) (Cotto et al. 2018), we identified the essential genes and drug availability for each gene.

### 2.9 Statistical Analysis

The R 4.3.1 program (http://www.Rproject.org) was used for all studies.

We used inverse variance weighting (IVW) (Burgess et al. 2013), MR–Egger (Bowden et al. 2015), weighted median (Bowden et al. 2016), weighted mode (Hartwig et al. 2017), and simple mode (Zhu et al. 2022) methods to evaluate the causal relationship between MS and 731 immune phenotypes. These algorithms were executed using the “MendelianRandomization” (v0.4.3) and “TwoSampleMR” packages in R. We utilized the MR‐PRESSO global test (Verbanck et al. 2018) and the MR–Egger regression method (Bowden et al. 2015) to account for and exclude any potential effects of horizontal pleiotropy. Simultaneously, we employed Cochran's *Q* statistics to assess the differences among various IVs (Hemani, Bowden, et al. 2018). Additionally, we performed a leave‐one‐out analysis to assess each SNP's unique influence on MR estimates (Burgess et al. 2017).

## 3 Results

### 3.1 Causal Effect From Multiple Immunophenotypes to MS

**FIGURE 1:**
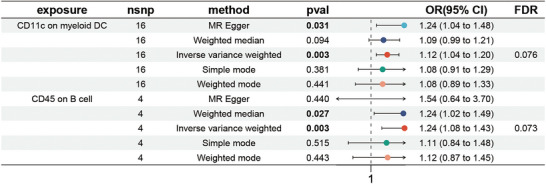
Causal relationships between MS and immune cell traits were shown by forest plots. MS, multiple sclerosis.

First, we employed the same methodology to investigate the effect of immunophenotypes on MS. After FDR adjustment (*p*_FDR_ < 0.05), we did not find any immunophenotype significantly associated with MS. However, when we relaxed the significance threshold to 0.10, two suggestive immunophenotypes emerged. These were CD11c on myeloid DC (odds ratio [OR] = 1.12, 95% CI = 1.04–1.20, *p* = 0.003, *p*_FDR_ = 0.076) (Figure 1 and Table S1) and CD45 on B cell (OR = 1.24, 95% CI = 1.08–1.43, *p* = 0.003, *p*_FDR_ = 0.073) (Figure 1 and Table S1). The other four MR techniques—MR–Egger, weighted median, simple mode, and weighted mode—all corroborated these relationships (Figure 1 and Figure S2). We further confirmed the robustness of these causal relationships through heterogeneity tests, horizontal pleiotropy assessments, and leave‐one‐out analyses (Figure S2). Visual representations such as scatter plots and funnel plots are available to illustrate the results (Figure S1).

### 3.2 Bayesian Colocalization Analysis for CD11c on Myeloid DC, CD45 on B Cells, and MS

To further elucidate the potential mechanisms governing the participation of CD11c on myeloid DC and CD45 on B cells in the initiation and progression of MS, moreover, to explore potential targets for intervention in MS, we examined the common causative SNPs between these two categories of immune cells and MS using COLOC analysis. Initially, we identified the two most significant SNPs and their chromosomal locations in immune cells based on their respective *p*‐values in GWAS studies. Subsequently, we conducted a colocalization study at the same genomic positions in the context of MS. Unfortunately, none of the PP.H4 readings exceeded 0.75, with a PP.H4 of 0.016 for CD45 on B cells and 0.65 for CD11c on myeloid DC (Figure S3 and Table S2). This limitation may stem from the inherent shortcomings of the colocalization approach; specifically, only positions near the SNP with the most significant *p*‐value (top SNP) are extracted and analyzed, resulting in a significant loss of data. Consequently, we adopted an alternative approach: the MAGMA gene.

### 3.3 Gene Screening for the Same Genetic Mechanism Underlying Two Immune Cells and MS

**FIGURE 2:**
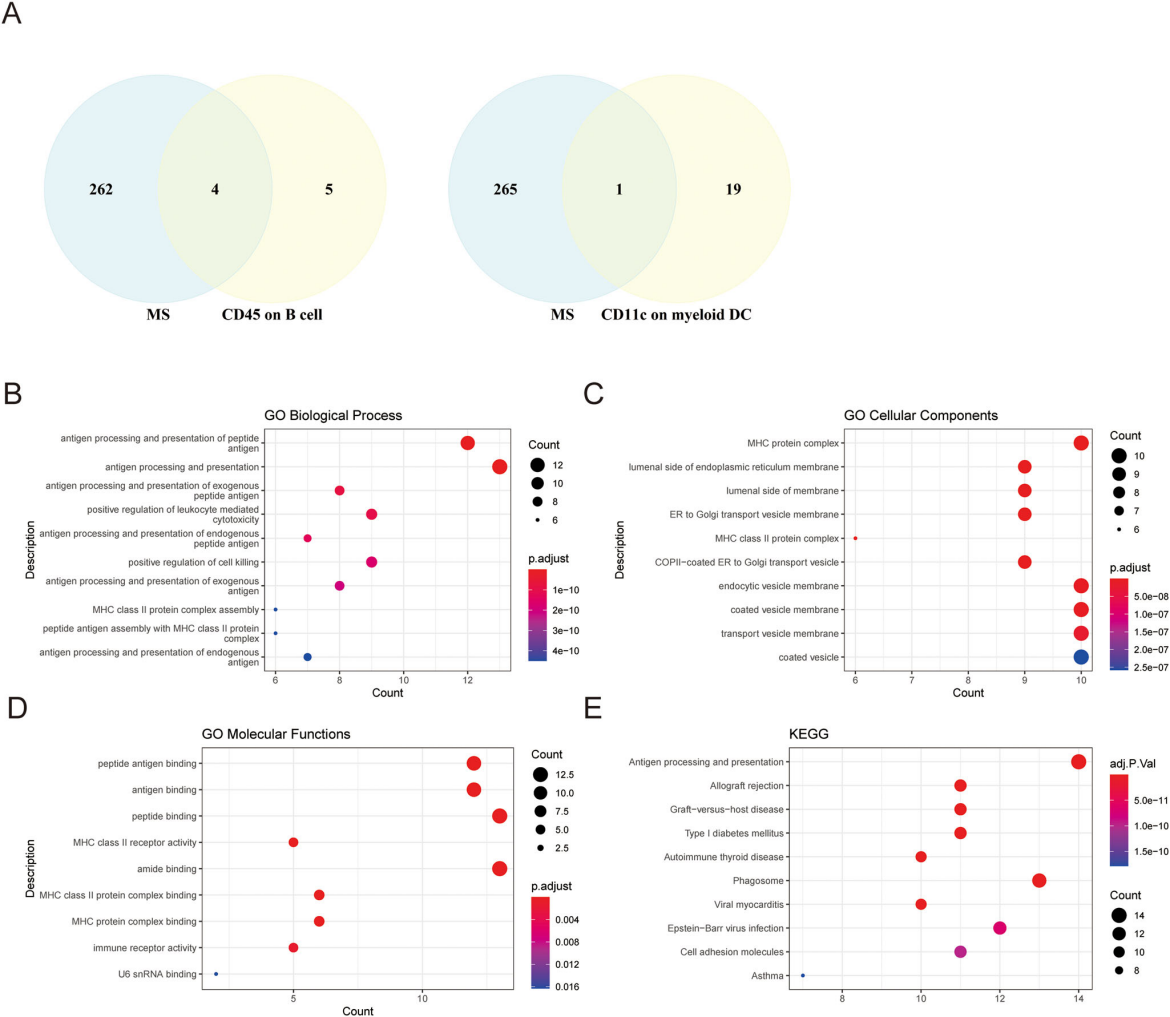
The MS‐related pleiotropic genes of CD45 on B cell and CD11c on myeloid DC discovered by MAGMA and PLACO, and the GO and KEGG functional enrichment analysis of these genes. (A) Venn diagram to show the number of shared genes (FDRMAGMA < 0.05) between the CD45 on B cell and MS, and CD11c on myeloid DC and MS. (B) GO biological process analysis of pleiotropic genes screened by PLACO. (C) GO cellular components analysis of pleiotropic genes screened by PLACO. (D) GO molecular functions analysis of pleiotropic genes screened by PLACO. (E) KEGG enrichment analysis of pleiotropic genes screened by PLACO.

To detect pleiotropic genes, we utilized MAGMA, which aggregates SNP‐level associations into single gene‐level association signals using summary statistics. In our research conducted at the Complex Trait Genetics Lab (https://ctg.cncr.nl/), we employed an annotation file that defines SNPs within specific genes. This allowed us to obtain the *p*‐value and *Z*‐statistic for each gene. Note that 20, 9, and 266 genes were found to be significantly linked (FDRMAGMA < 0.05) with CD11c on myeloid DC, CD45 on B cell, and MS by MAGMA analysis, respectively. The FDRMAGMA value sorting indicates that genes such as CD11b, CD11c, serine protease, and antiapoptotic proteins are present in CD11c on myeloid DC. Members of the cycling deaminase gene family, Chromosome 6 open reading framework 15, and so forth are primarily included in CD45 on B cell. Decapping exonuclease, ribonuclease, acyltransferase, human leukocyte antigen, and so forth are included in MS (Figure 2A and Table S3).

Using *Z*‐statistics derived from MAGMA, we conducted PLACO analysis to identify pleiotropic genes. Each gene underwent scrutiny by PLACO to ascertain potential pleiotropic connections between two immune cell types and MS. As a result, we identified 56 genes (FDRPLACO < 0.05) that exhibited simultaneous associations with CD45 on B cell and MS. Notable genes in this set included *C6orf15*, *PSORS1C1*, *HLA‐DQA1*, *PSORS1C2*, and *ATP6V1G2*, among others. Additionally, we uncovered five pleiotropic genes (FDRPLACO < 0.05) concurrently linked to CD11c on myeloid DC and MS, such as *MUC21*, *LY6G6C*, *SYNGAP1*, *GPSM3*, and *WDR46* (Table S4). These identified genes hold significant potential to serve as the molecular foundation underlying the involvement of these two immune cells in the pathogenesis of MS.

It is noteworthy that the gene sets identified by MAGMA and PLACO were not entirely overlapping, which is expected given their methodological differences. MAGMA is optimized for detecting cumulative polygenic effects across multiple variants within a gene, whereas PLACO focuses on pleiotropy at the locus level and is more sensitive to shared causal variants between traits. Therefore, the findings from the two approaches should be regarded as complementary, providing converging but distinct perspectives on the genetic mechanisms linking immune cells to MS.

### 3.4 Functional Analysis for Pleiotropic Genes

To evaluate the enriched functions of pleiotropic genes, we first conducted GO and KEGG enrichment analyses for these 61 genes (56 genes for CD45 on B cell and MS, and five genes for CD11c on myeloid DC and MS). In terms of GO biological processes, these pleiotropic genes showed enrichment in various areas, including polypeptide antigens, exogenous peptide antigens, endogenous peptide antigens, exogenous antigens, endogenous antigen processing and presentation, leukocyte‐mediated cytotoxicity, and positive regulation of cell killing. Additionally, a small number of genes were found to be enriched in MHC Class II protein complex assembly and peptide antigen processing (Figure 2B). Regarding GO cellular components, these pleiotropic genes were enriched in processes such as vesicle transport, endocytosis, and vesicle encapsulation. They were also found to be associated with the MHC protein complex, luminal side of the endoplasmic reticulum membrane, luminal side of the membrane, and MHC Class II protein complex (Figure 2C). As for GO molecular functions, these pleiotropic genes showed enrichment in peptide antigen, antigen, peptide, amide, MHC Class II protein complex, and MHC protein complex binding. Furthermore, there was evidence of activation of MHC Class II receptors and immune receptors (Figure 2D).

According to the KEGG enrichment analysis, these 61 genes were found to be significantly enriched in multiple pathways. The most significant pathway among them was antigen processing and presentation. Additionally, they exhibited enrichment in pathways such as allograft rejection, graft versus host disease, Type I diabetes mellitus, autoimmune thyroid disease, phagosome, viral myocarditis, Epstein–Barr virus (EBV) infection, cell adhesion molecules, and asthma (Figure 2E).

### 3.5 Tissue‐Specific Analyses for Pleiotropic Genes

**FIGURE 3:**
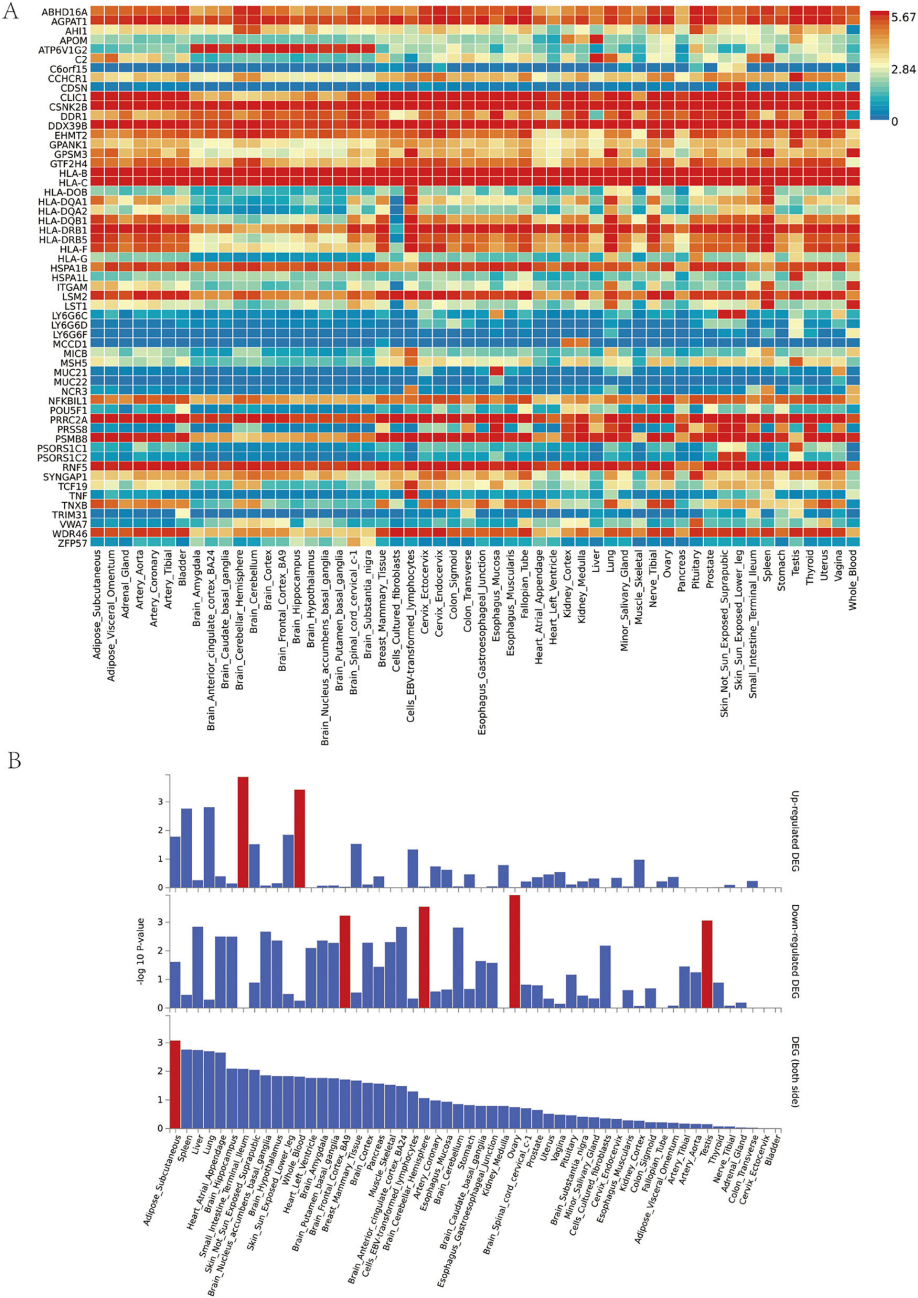
Tissue‐specific analysis of 61 genes. (A) Gene expression heatmap across GTEx v8 54 tissue types and (B) differentially expressed genes across GTEx v8 54 common tissue types.

To discern genes characterized by brain‐specific expression and pinpoint those most strongly associated with demyelinating lesions in MS, we conducted additional tissue‐specific analyses for pleiotropic genes. In brain tissue, we noted distinct expression patterns for five genes: *ATP6V1G2, HLA‐DQB1, HLA‐DRB1, HLA‐DRB5*, and *WDR46* (see Figure 3A). Enrichment analysis across 54 commonly studied tissues revealed significant upregulation of these genes in intestinal and blood tissues, coupled with downregulation in brain tissue, ovaries, and testes (refer to Figure 3B). This observed downregulation of pleiotropic genes in brain tissue lends support to the credibility of our finding.

### 3.6 PPI, Druggability Evaluation, and HLA Sensitivity Analysis

**FIGURE 4:**
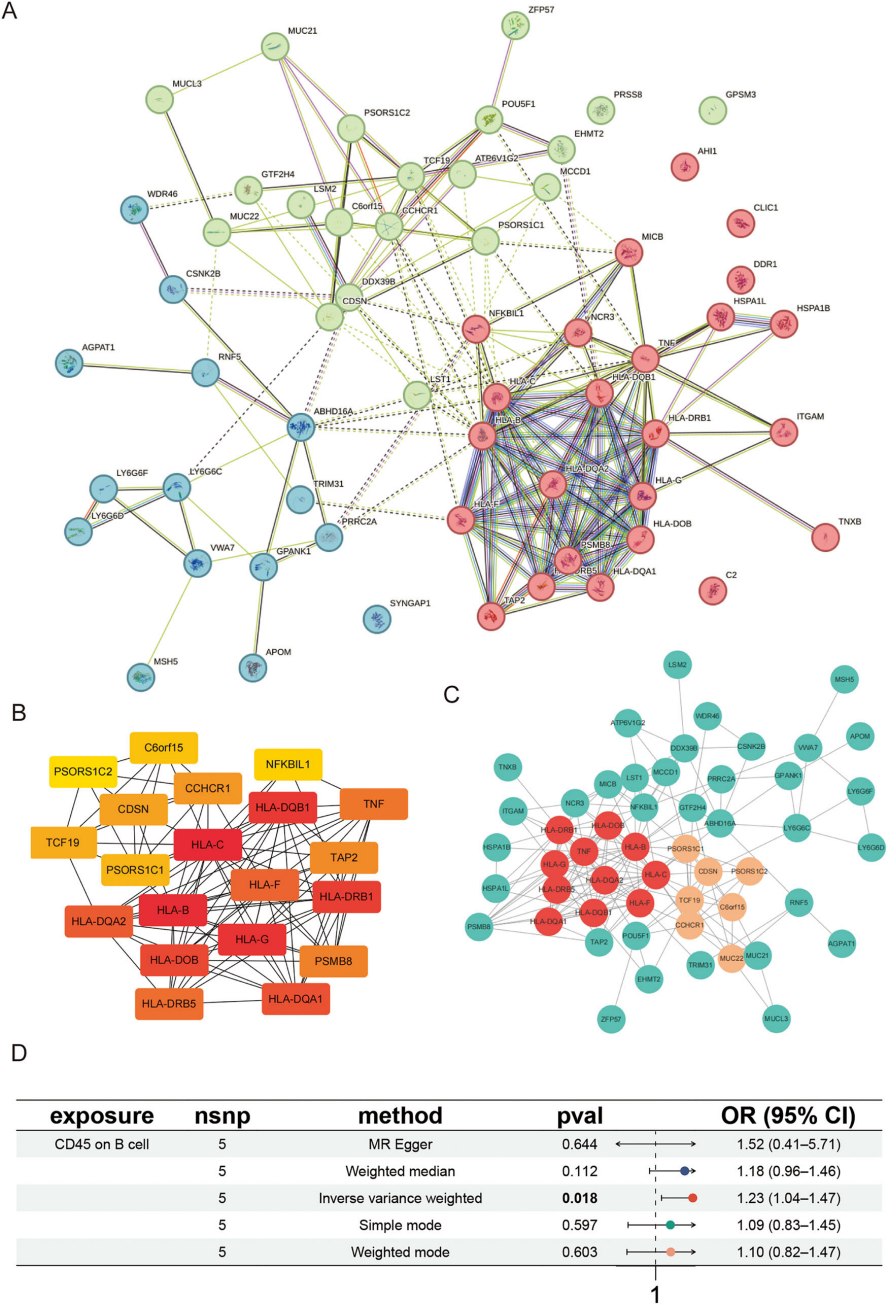
(A–C) Protein–protein interaction enrichment analysis by STRING database (https://string‐db.org/). (D) Causal relationship between CD45 on B cells and MS after excluding HLA‐related SNPs, displayed in forest plots.

Finally, the PPI analysis of the set of 61 pleiotropic genes was also performed, using the STRING database (https://string‐db.org/), to reveal further details of this most critical PPI and identify the key protein involved in the MS. Our analysis revealed significant associations among several molecules, particularly the HLA proteins. Notably, the central protein in the network was identified as HLA‐DQA2, which exhibited interactions with TNF (Figure 4A). Using MCODE and Cytohubba in Cytoscape, we identified key proteins in the protein‐interaction network: Module I centered on HLA‐DQA2 and TNF, and another module centered on C6orf15 (Figure 4B) and the key top 20 proteins containing HLA‐C, HLA‐B, and HLA‐G (Figure 4C).

Given the PPI analysis found limited interactions between the identified potential causal proteins, we used DGIdb to prioritize the potential druggable targets by integrating information from drug–gene interactions. Among the key top 20 proteins identified by PPI, there were 14 proteins with possible target drugs. Among them, there are more targeted drugs for HLA‐like molecules and TNF (Table S5).

To assess the influence of HLA variation on the causal relationship between immune cell phenotypes and MS, we conducted a sensitivity MR analysis, excluding the HLA‐related SNP. Notably, no HLA‐related SNPs were identified in the IVs for CD11c on myeloid dendritic cells (DCs), whereas one HLA‐related SNP was present in the IVs for CD45 on B cells. After exclusion, the association between CD45 on B cells and MS remained directionally consistent, though the effect size was attenuated (OR = 1.23, *p* = 0.018 compared to *p* = 0.003 before exclusion) (Figure 4D).

### 3.7 Causal Effect From MS to Immunophenotypes

**FIGURE 5:**
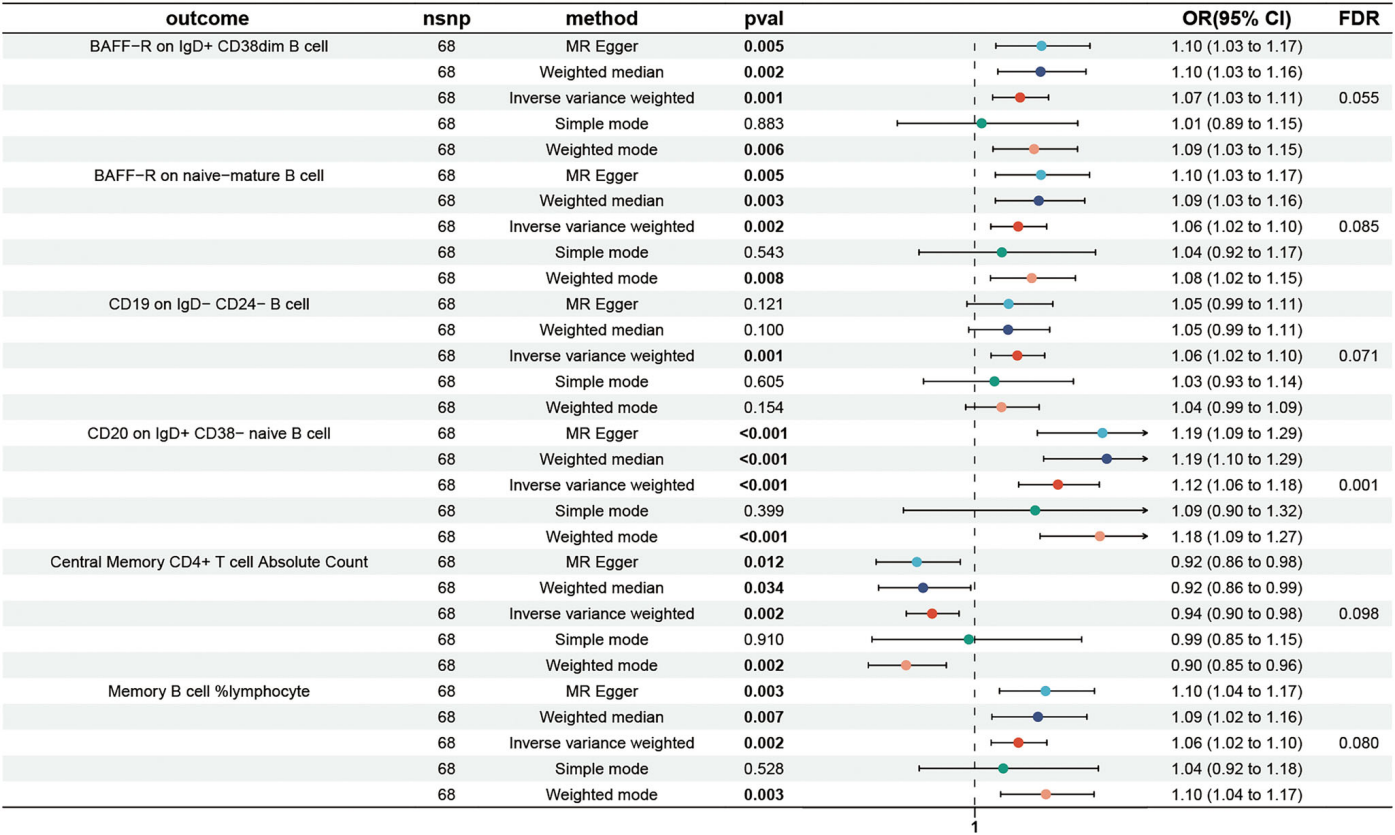
Causal relationships between immune cell traits and MS were shown by forest plots. MS, multiple sclerosis.

We looked into how MS affected immunological phenotypes using a dual‐sample MR approach. The main analytical method used was the IVW method, and we modified the *p*‐values using the FDR method. At the conventional significance level (*p* = 0.05), we identified only one B cell subtype. Consequently, we raised the significance threshold to 0.1, leading to the identification of six immune phenotypes—one within T lymphocytes and the remaining five within B lymphocytes. In the presence of MS, we observed an increase in the expression of B‐cell activating factor receptor (BAFF‐R) on IgD^+^CD38^dim^ B cells (OR = 1.07, 95% CI = 1.03–1.11, *p* = 0.001, *p*_FDR_ = 0.055) (Figure 5 and Table S6) and naive mature B cells (OR = 1.06, 95% CI = 1.02–1.10, *p* = 0.002, *p*_FDR_ = 0.085) (Figure 5 and Table S6). Furthermore, CD19 on IgD^−^CD24^−^ B cells (OR = 1.06, 95% CI = 1.02–1.10, *p* = 0.001, *p*_FDR_ = 0.071) (Figure 5 and Table S6) and CD20 on IgD^+^CD38^−^ naive B cells (OR = 1.12, 95% CI = 1.06–1.18, *p* < 0.001, *p*_FDR_ = 0.001) (Figure 5 and Table S6) also exhibited increases, as did memory B cell %lymphocytes (OR = 1.06, 95% CI = 1.02–1.10, *p* = 0.002, *p*_FDR_ = 0.080) (Figure 5 and Table S6). On the other hand, central memory CD4^+^ T cell absolute count (OR = 0.94, 95% CI = 0.90–0.98, *p* = 0.098, *p*_FDR_ = 0.080) (Figure 5 and Table S6) showed a decrease. The other four MR techniques—MR–Egger, weighted median, simple mode, and weighted mode—all supported these relationships (Figure 5 and Figure S4). Subsequently, we assessed the robustness of these causal relationships through heterogeneity tests, horizontal pleiotropy assessments, and leave‐one‐out analysis (Figure S5). Visualizations in the form of scatter plots and funnel plots were employed to illustrate the results (Figure S6, S7).

## 4 Discussion

MS is a complex autoimmune disease marked by lifelong disability, with its precise etiology remaining elusive. Despite genetic and epidemiological investigations providing valuable insights, establishing a clear causal relationship between immune cells and MS pathogenesis has remained a challenge. In this study, we identified causal associations between MS and immune cell phenotypes, specifically B lymphocytes and DCs, offering new perspectives on the immunological mechanisms underlying MS.

### 4.1 Causal Links Between Immune Cells and MS

Using MR analysis, we confirmed that increased levels of CD45 on B cells and CD11c on myeloid DCs were associated with an elevated risk of MS, which aligns with previous studies (Asashima et al. 2022; Liu et al. 2022). B cells contribute to MS onset and progression through antigen presentation, cytokine production, and antibody generation, while DCs activate autoreactive T cells, promoting inflammation (Yang et al. 2021; Mailaender et al. 2025; Li et al. 2024). These findings further support the efficacy of B cell–targeted therapies in MS, such as B cell inhibitors, which have shown promise in clinical settings (Montalban et al. 2019; Steinman et al. 2022; Hauser et al. 2020; Svenningsson et al. 2022).

Our study also emphasizes the role of B cells in antigen presentation, which may disrupt self‐immune tolerance and affect the interactions between antigen‐presenting cells (APCs) and T cells. This observation aligns with clinical findings that EBV)‐infected B cells accumulate in MS lesions, particularly in intrameningeal follicles and white matter lesions (Orr and Steinman 2025). Enrichment analyses further confirmed the central role of B cells in MS, suggesting that dysregulation of antigen‐presenting pathways could exacerbate the disease, highlighting the need for targeted therapeutic strategies.

### 4.2 Mechanistic Insights Into Immune Cell Contributions to MS

To delve deeper into the molecular mechanisms underlying immune cell involvement in MS, we employed advanced analytical techniques, including Bayesian colocalization and MAGMA software (de Leeuw et al. 2015). While Bayesian colocalization analysis focused on SNP loci did not yield significant results, MAGMA's gene annotation revealed 61 pleiotropic genes connected to B cell and DC functions. These findings suggest that MS development is underpinned by complex genetic interactions involving these immune cell types. Notably, genes involved in RNA metabolism (e.g., decapping exonuclease and ribonuclease) and lipid metabolism (e.g., acyltransferase) were identified, shedding light on the cellular processes disrupted in MS. This aligns with recent studies implicating RNA metabolism in MS pathophysiology and provides new avenues for understanding the molecular basis of the disease (Wolozin and Ivanov 2019; Salapa et al. 2024).

### 4.3 Role of HLA and Pleiotropic Genes in MS

Through PLACO analysis (Ray and Chatterjee 2020we identified significant pleiotropic genes associated with MS, including PSORS1C1, PSORS1C2, and HLA‐DQA1. These genes have been previously linked to autoimmune diseases and highlight the genetic complexity contributing to MS susceptibility. Additionally, SYNGAP1, a gene related to synapse function, was identified as pleiotropically associated with DCs and MS (Zeng et al. 2016; Bednarczuk et al. 2024), reinforcing the importance of immune cell interactions in disease progression. HLA genes, particularly HLA‐DQB1, HLA‐DRB1, and HLA‐DRB5, emerged as central to MS pathogenesis (Wang et al. 2020; Barrie et al. 2024). The expression patterns of these HLA genes validate their critical role in MS and especially explain the prior findings of ethnic variability in MS prevalence (Barrie et al. 2024). With ongoing research into HLA‐targeted interventions across various diseases, including autoimmune disorders and cancer (Pearlman et al. 2021), these results position HLA as a new promising therapeutic target in MS.

While HLA genes are central to MS, our sensitivity analysis underscores the disease's complex genetic basis. Excluding the HLA‐related SNP attenuated but did not eliminate the association between CD45 on B cells and MS, indicating that HLA variation strongly shapes this link but is not the sole determinant. These results highlight the multifactorial nature of MS, involving the interplay of HLA variation, other genetic factors, immune cell function, and environmental influences.

### 4.4 Immune Cell Subgroup Dysregulation Post‐MS Onset

In addition to genetic insights, we examined immune cell alterations following the onset of MS. Our data revealed distinct changes in immune cell populations, particularly within B cell subtypes. Elevated expression of BAFF‐R, a key molecule for B cell activation, was observed in IgD+CD38dim and naive mature B cells, which are crucial for B cell differentiation and survival (Möckel et al. 2021). This observation suggests that heightened BAFF signaling may contribute to autoimmune activation in MS. Additionally, increased expression of CD19 on IgD−CD24− B cells and CD20 on IgD+CD38− naive B cells highlights the role of specific B cell subtypes in disease progression. In contrast, a reduction in central memory CD4+ T cells was observed, consistent with prior clinical studies. These findings underscore the importance of monitoring immune cell subgroups as potential diagnostic markers for MS, offering insights into immune system activation and disease progression.

### 4.5 Limitations and Future Directions

While our study provides substantial insights into the genetic and immunological mechanisms underlying MS, there are several limitations to consider. First, the GWAS data used were limited to the European population, and further research including diverse ethnic groups is necessary to expand the applicability of these findings. Second, the interactions between immune cells, which could influence our results, were not explored in detail. Future studies should aim to elucidate these interactions. In addition, we recognize that relaxing the FDR cutoff in MR for certain suggestive associations could potentially elevate the false positive rate. Finally, the lack of clinical data on MS subtypes and disease severity limited our ability to investigate the relationship between immune cell alterations and MS progression in clinical settings.

## 5 Conclusion

In summary, our study offers critical insights into the genetic and immunological foundations of MS. By establishing causal links between immune cell phenotypes—specifically B lymphocytes and DCs—and MS, we identified 61 pleiotropic genes associated with the disease. Our findings underscore the significant role of B cells in antigen processing and presentation, with a particular focus on HLA genes as potential therapeutic targets. This research not only enhances our understanding of MS pathogenesis but also provides novel biomarkers for diagnosis and highlights potential avenues for therapeutic intervention. As such, our study paves the way for future research into immune cell‐targeted therapies and diagnostic strategies in MS.

## Author Contributions

X.L. and X.W. designed the study. Y.W. and X.W. collected and analyzed the data. X.W. and X.L. wrote the manuscript. All authors contributed to the article and approved the submitted version.

This work was supported by the National Natural Science Foundation of China (81900497, to Xiaojing Liu).

## Ethics Statement

The authors have nothing to report.

## Conflicts of Interest

The authors declare no conflicts of interest.

## Supporting information




**Supplementary Figure 1**: Scatter plots and Funnel plots of the immune cell traits on MS. (A) Scatter plot of CD45 on B cell; (B) Scatter plot of CD11c on myeloid DC; (C) Funnel plot of CD45 on B cell; (D) Funnel plot of CD11c on myeloid DC.

**Supplementary Figure 2**: Forest plots for leave‐one‐out plot and MR of the immune cell traits on MS. (A) Forest plot of CD45 on B cell; (B) Forest plot of CD11c on myeloid DC; (C) Forest plot for leave‐one‐out plot of CD45 on B cell; (D) Forest plot for leave‐one‐out plot of CD11c on myeloid DC.

**Supplementary Figure 3**: Colocalization analysis depicted genomic regions and causal SNPs associated with MS in CD45 on B cell and CD11c on myeloid DC. (A) Colocalization results of CD45 on B cell and MS; (B) Colocalization results of CD11c on myeloid DC and MS.

**Supplementary Figure 4**: Forest plots of MS on the immune cell traits. (A) Central Memory CD4+ T cell Absolute Count; (B) BAFF‐R on IgD+ CD38dim B cell; (C) Memory B cell %lymphocyte; (D) BAFF‐R on naive‐mature B cell; (E) CD19 on IgD‐ CD24‐ B cell; (F) CD20 on IgD+ CD38‐ naive B cell. MS: Multiple sclerosis.

**Supplementary Figure 5**: Forest plots for leave‐one‐out plot of MS on the immune cell traits. (A) Central Memory CD4+ T cell Absolute Count; (B) BAFF‐R on IgD+ CD38dim B cell; (C) Memory B cell %lymphocyte; (D) BAFF‐R on naive‐mature B cell; (E) CD19 on IgD‐ CD24‐ B cell; (F) CD20 on IgD+ CD38‐ naive B cell. MS: Multiple sclerosis.

**Supplementary Figure 6**: Scatter plots of MS on the immune cell traits. (A) Central Memory CD4+ T cell Absolute Count; (B) BAFF‐R on IgD+ CD38dim B cell; (C) Memory B cell %lymphocyte; (D) BAFF‐R on naive‐mature B cell; (E) CD19 on IgD‐ CD24‐ B cell; (F) CD20 on IgD+ CD38‐ naive B cell. MS: Multiple sclerosis.

**Supplementary Figure 7**: Funnel plots of MS on the immune cell traits. (A) Central Memory CD4+ T cell Absolute Count; (B) BAFF‐R on IgD+ CD38dim B cell; (C) Memory B cell %lymphocyte; (D) BAFF‐R on naive‐mature B cell; (E) CD19 on IgD‐ CD24‐ B cell; (F) CD20 on IgD+ CD38‐ naive B cell. MS: Multiple sclerosis.

**Supplementary Table 1**: brb371292‐sup‐0008‐TableS1.xlsx

**Supplementary Table 2**: brb371292‐sup‐0009‐TableS2.xlsx

**Supplementary Table 3**: brb371292‐sup‐0010‐TableS3.xlsx

**Supplementary Table 4**: brb371292‐sup‐0011‐TableS4.xlsx

**Supplementary Table 5**: brb371292‐sup‐0012‐TableS5.xlsx

**Supplementary Table 6**: brb371292‐sup‐0013‐TableS6.xlsx

## Data Availability

The data that support the findings of this study are available in GWAS at: https://www.ebi.ac.uk/gwas/. These data were derived from the resources available in the public domain.
