# Supplementary figures and images for: Elucidating the Causal Relationships Between B Cells, Dendritic Cells, and Multiple Sclerosis Pathogenesis
Source: Brain Behav. 2026 Feb 28;16(3):e71292. doi: 10.1002/brb3.71292 (PMC12949720; doi:10.1002/brb3.71292)

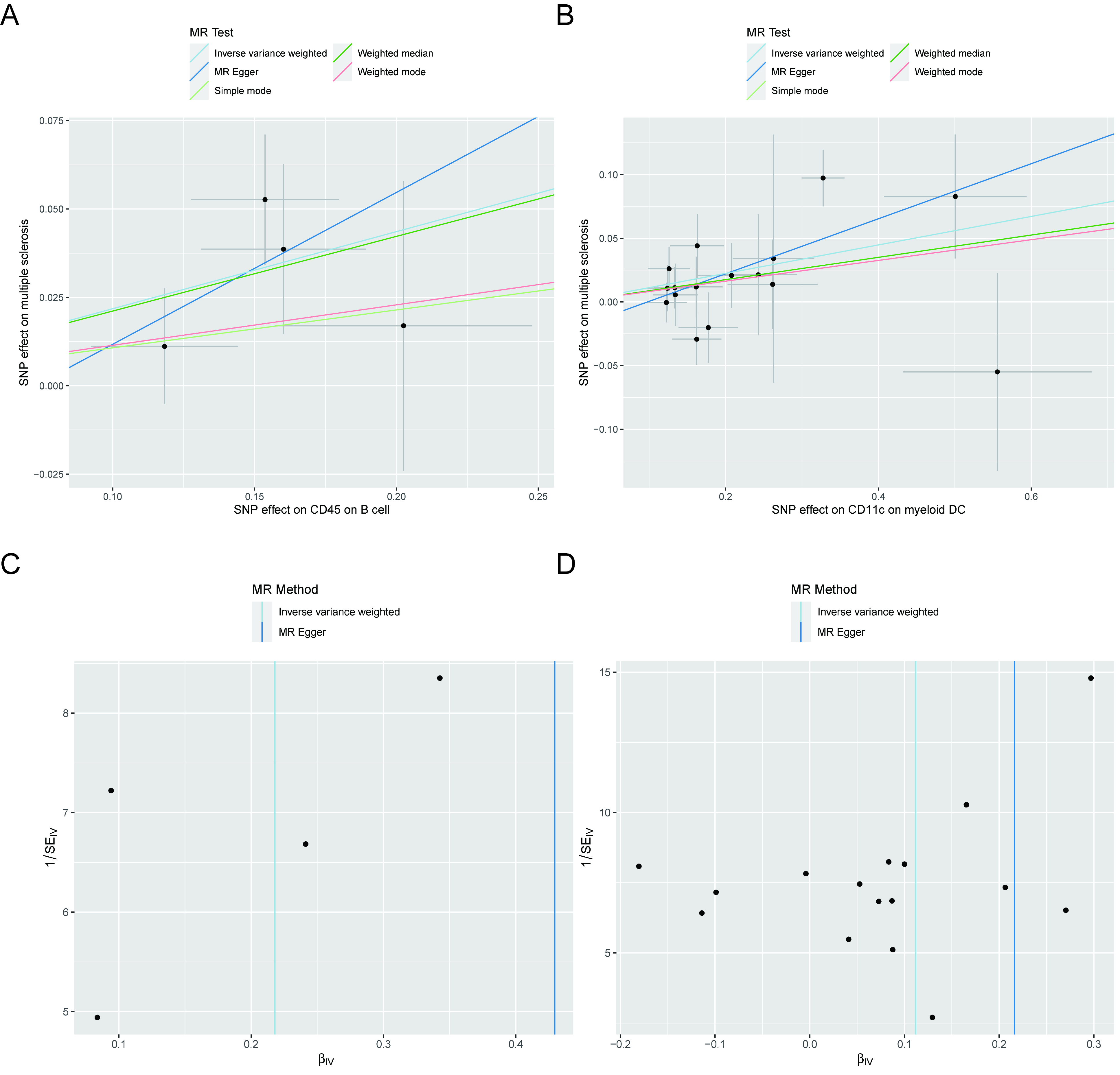

Supplement: Supplementary file 1 — Supplementary Figure 1: Scatter plots and Funnel plots of the immune cell traits on MS. (A) Scatter plot of CD45 on B cell; (B) Scatter plot of CD11c on myeloid DC; (C) Funnel plot of CD45 on B cell; (D) Funnel plot of CD11c on myeloid DC. [file BRB3-16-e71292-s001.tif]

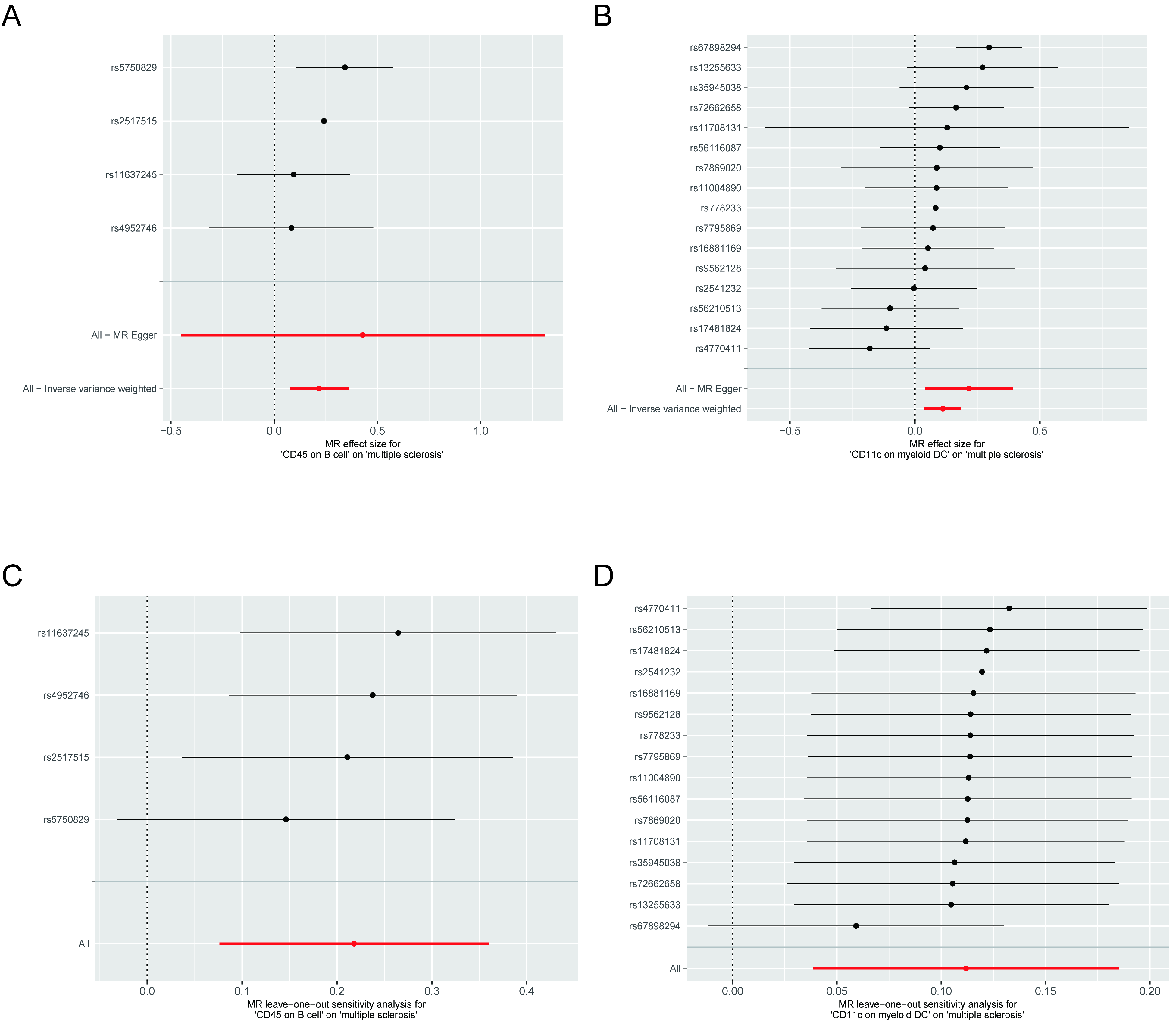

Supplement: Supplementary file 2 — Supplementary Figure 2: Forest plots for leave‐one‐out plot and MR of the immune cell traits on MS. (A) Forest plot of CD45 on B cell; (B) Forest plot of CD11c on myeloid DC; (C) Forest plot for leave‐one‐out plot of CD45 on B cell; (D) Forest plot for leave‐one‐out plot of CD11c on myeloid DC. [file BRB3-16-e71292-s012.tif]

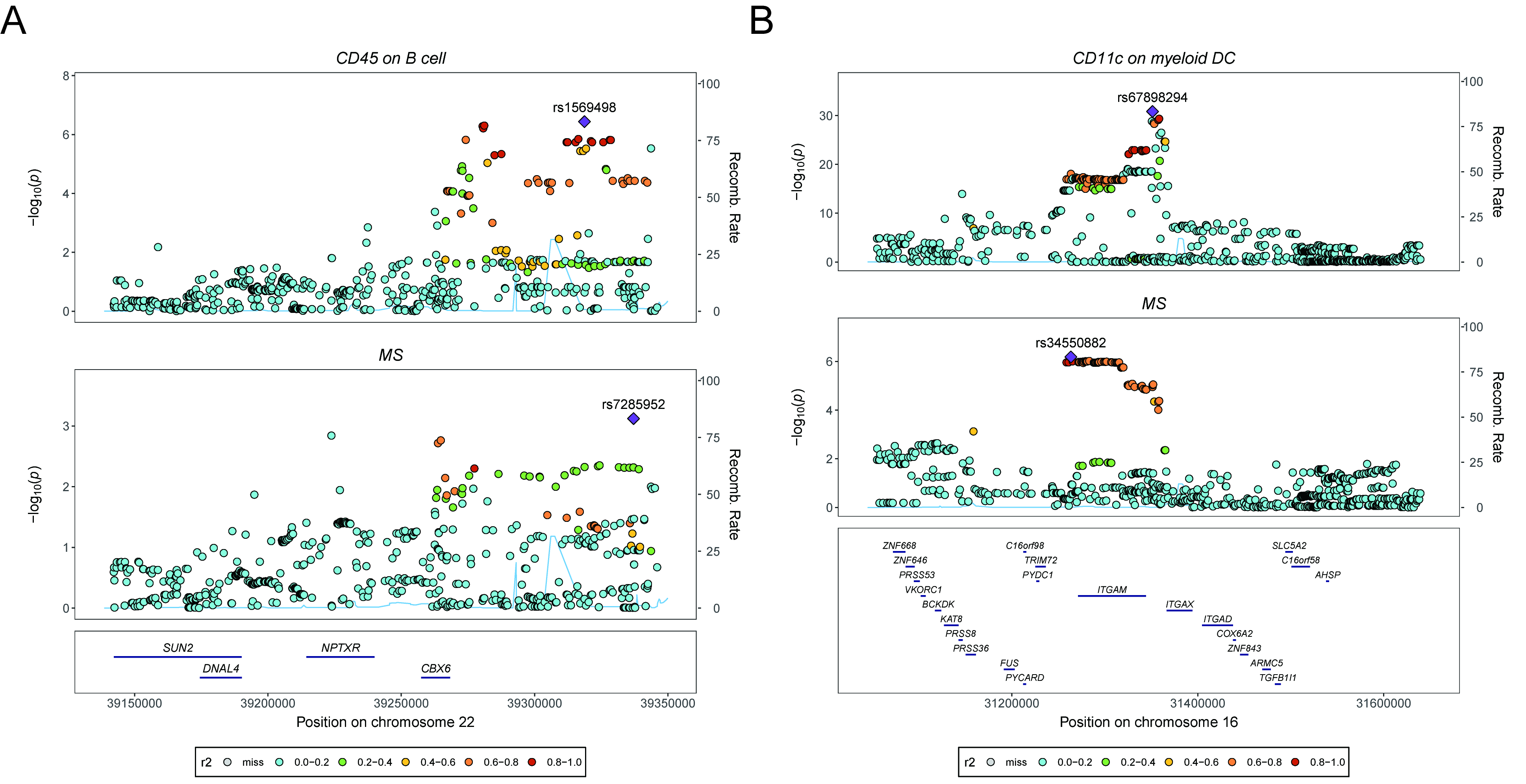

Supplement: Supplementary file 3 — Supplementary Figure 3: Colocalization analysis depicted genomic regions and causal SNPs associated with MS in CD45 on B cell and CD11c on myeloid DC. (A) Colocalization results of CD45 on B cell and MS; (B) Colocalization results of CD11c on myeloid DC and MS. [file BRB3-16-e71292-s005.tif]

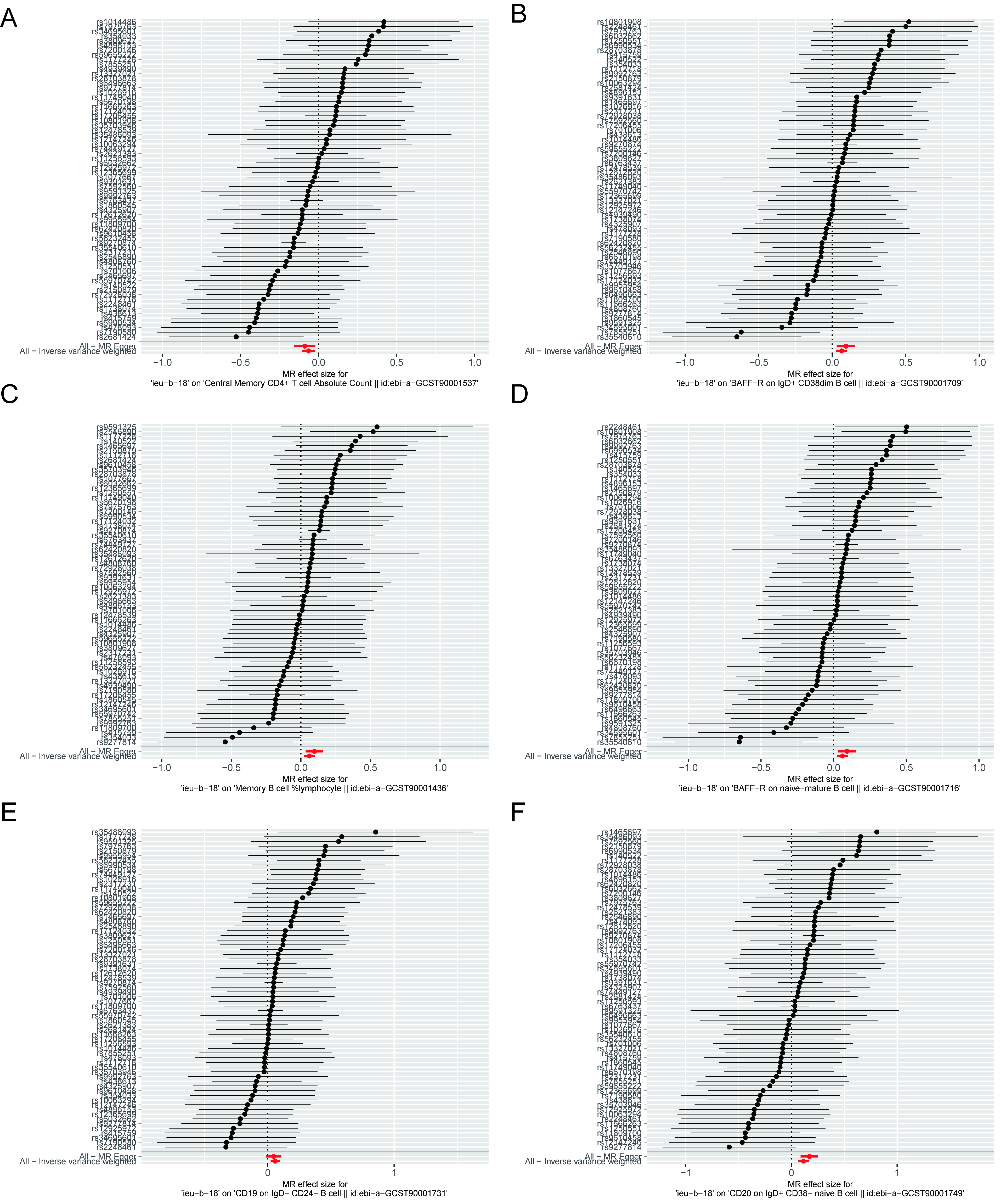

Supplement: Supplementary file 4 — Supplementary Figure 4: Forest plots of MS on the immune cell traits. (A) Central Memory CD4+ T cell Absolute Count; (B) BAFF‐R on IgD+ CD38dim B cell; (C) Memory B cell %lymphocyte; (D) BAFF‐R on naive‐mature B cell; (E) CD19 on IgD‐ CD24‐ B cell; (F) CD20 on IgD+ CD38‐ naive B cell. MS: Multiple sclerosis. [file BRB3-16-e71292-s011.tif]

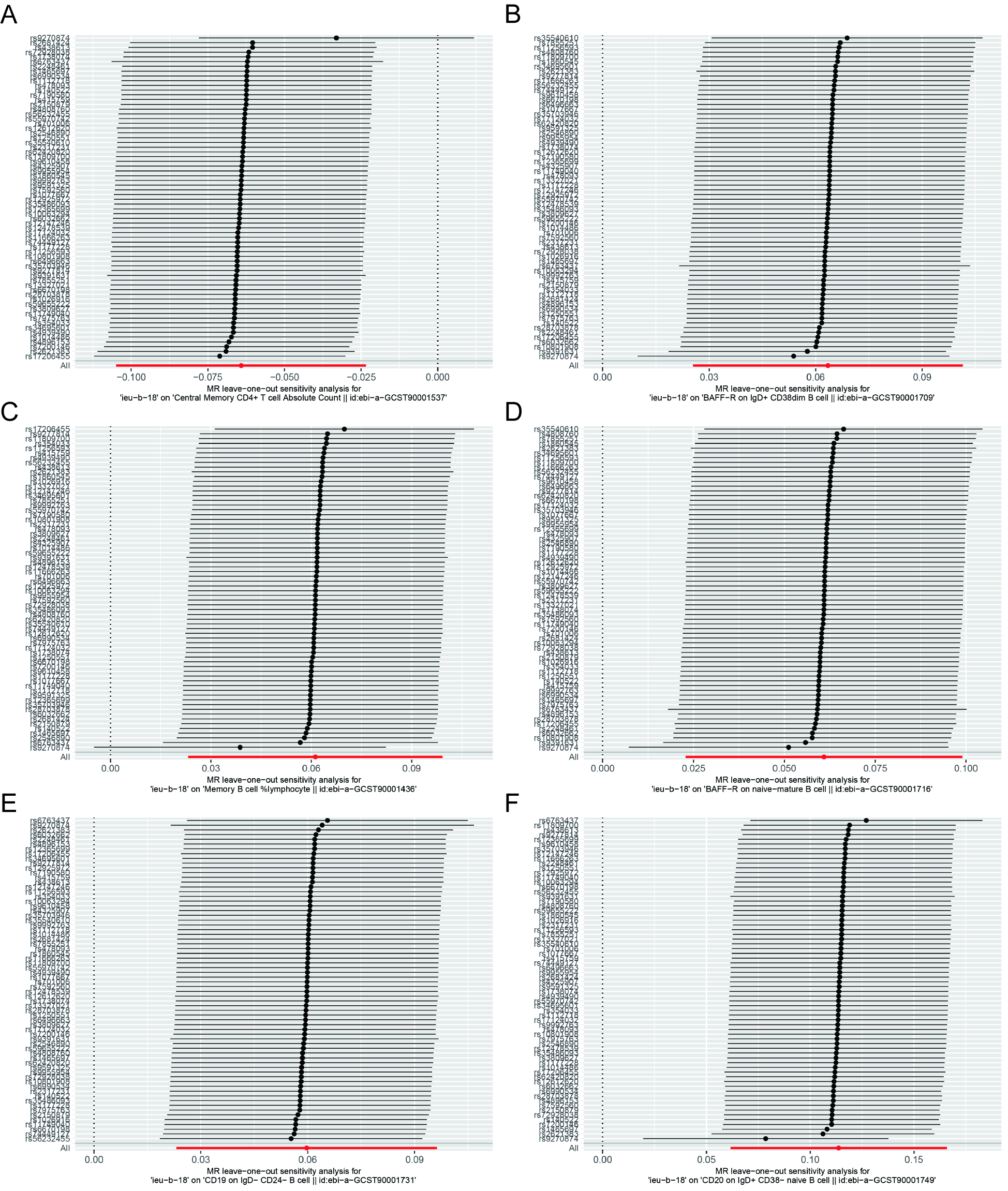

Supplement: Supplementary file 5 — Supplementary Figure 5: Forest plots for leave‐one‐out plot of MS on the immune cell traits. (A) Central Memory CD4+ T cell Absolute Count; (B) BAFF‐R on IgD+ CD38dim B cell; (C) Memory B cell %lymphocyte; (D) BAFF‐R on naive‐mature B cell; (E) CD19 on IgD‐ CD24‐ B cell; (F) CD20 on IgD+ CD38‐ naive B cell. MS: Multiple sclerosis. [file BRB3-16-e71292-s009.tif]

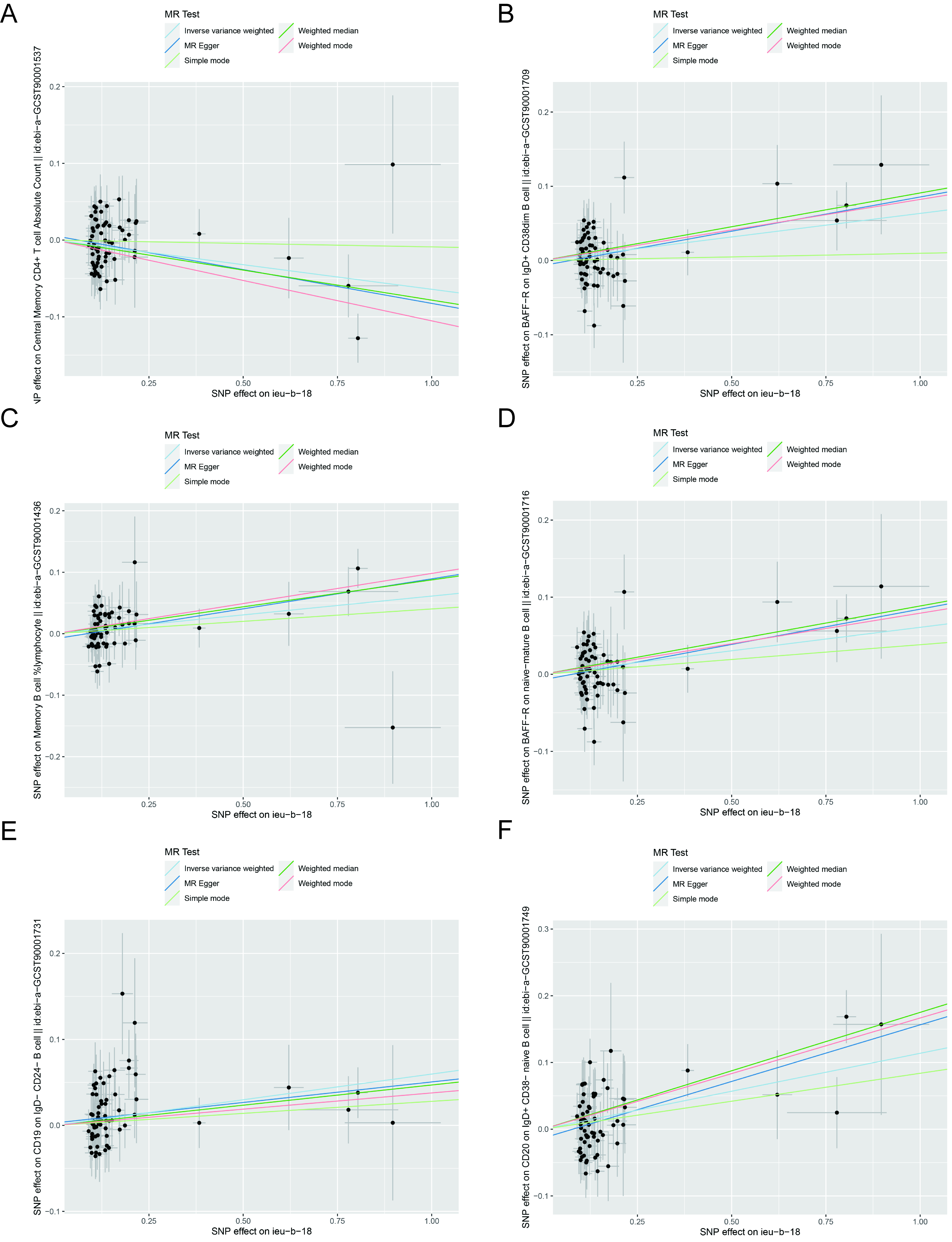

Supplement: Supplementary file 6 — Supplementary Figure 6: Scatter plots of MS on the immune cell traits. (A) Central Memory CD4+ T cell Absolute Count; (B) BAFF‐R on IgD+ CD38dim B cell; (C) Memory B cell %lymphocyte; (D) BAFF‐R on naive‐mature B cell; (E) CD19 on IgD‐ CD24‐ B cell; (F) CD20 on IgD+ CD38‐ naive B cell. MS: Multiple sclerosis. [file BRB3-16-e71292-s002.tif]

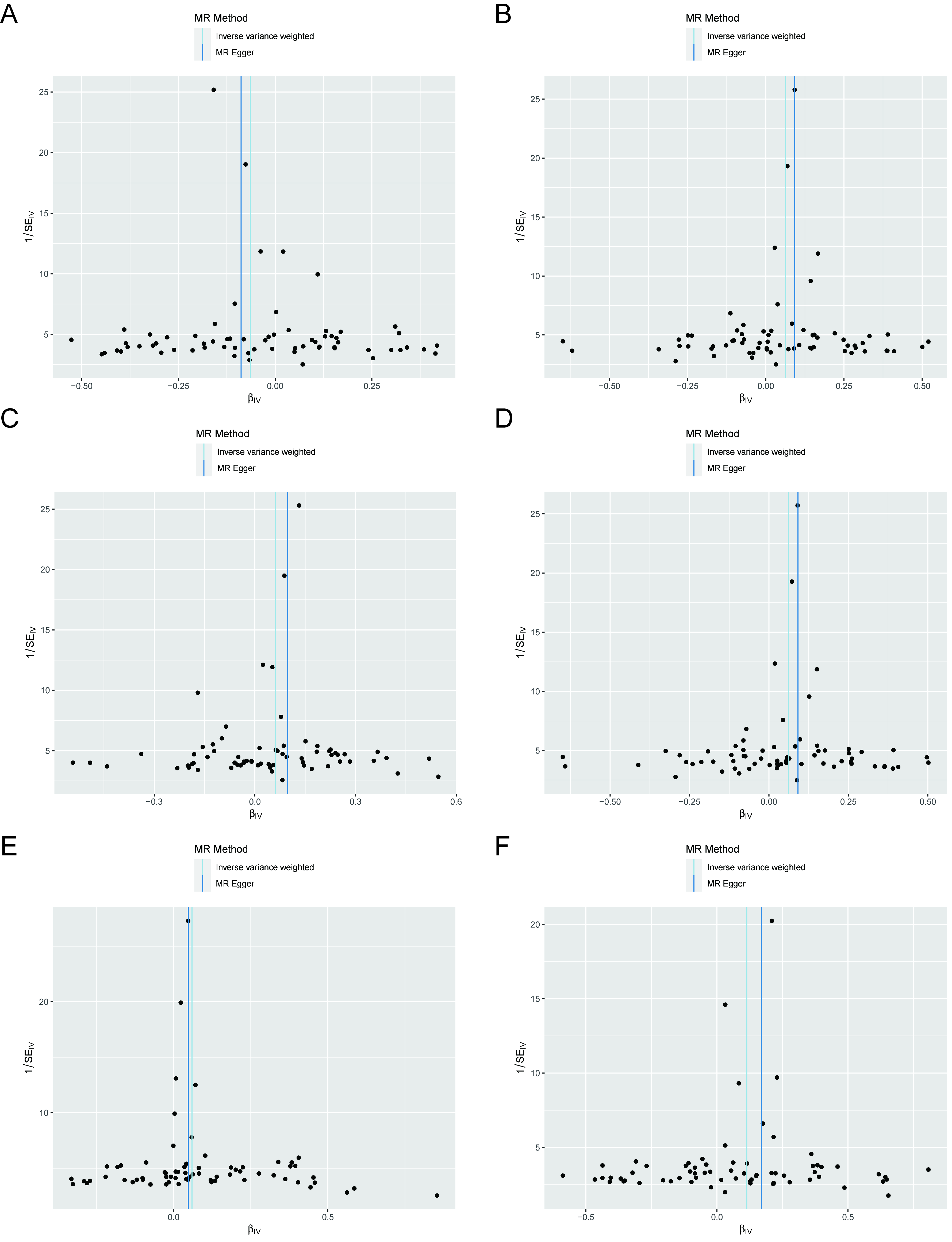

Supplement: Supplementary file 7 — Supplementary Figure 7: Funnel plots of MS on the immune cell traits. (A) Central Memory CD4+ T cell Absolute Count; (B) BAFF‐R on IgD+ CD38dim B cell; (C) Memory B cell %lymphocyte; (D) BAFF‐R on naive‐mature B cell; (E) CD19 on IgD‐ CD24‐ B cell; (F) CD20 on IgD+ CD38‐ naive B cell. MS: Multiple sclerosis. [file BRB3-16-e71292-s006.tif]
